# Safety of Non-vitamin K Antagonist Oral Anticoagulants on Outcomes After Transcatheter Aortic Valve Replacement: An Updated Meta-Analysis

**DOI:** 10.7759/cureus.72267

**Published:** 2024-10-24

**Authors:** Aman Goyal, Mahammed Khan Suheb, Humza Saeed, Mah I Kan Changez, Muhammad Daoud Tariq, Viraj Shah, Fatima Q Abbasi, Laveeza Fatima, Hritvik Jain, Sidhartha G Senapati, Vamsikalyan Borra

**Affiliations:** 1 Department of Internal Medicine, Seth Gordhandas Sunderdas (GS) Medical College and King Edward Memorial (KEM) Hospital, Mumbai, IND; 2 Department of Critical Care Medicine, Aurora St. Luke's Medical Center, Milwaukee, USA; 3 Department of Internal Medicine, Rawalpindi Medical University, Rawalpindi, PAK; 4 Department of Cardiothoracic Surgery, Yale University, New Haven, USA; 5 Department of Internal Medicine, Foundation University Medical College, Islamabad, PAK; 6 Department of Cardiology, Medical College of Georgia, Augusta University, Augusta, USA; 7 Department of Internal Medicine, Pakistan Institute of Medical Sciences, Islamabad, PAK; 8 Department of Internal Medicine, Allama Iqbal Medical College, Lahore, PAK; 9 Department of Internal Medicine, All India Institute of Medical Sciences, Jodhpur, Jodhpur, IND; 10 Department of Internal Medicine, Texas Tech University Health Sciences Center El Paso, El Paso, USA; 11 Department of Internal Medicine, The University of Texas Rio Grande Valley, Edinburg, USA

**Keywords:** cardiovascular outcomes, direct oral anticoagulants, meta-analysis, transcatheter aortic valve replacement, vitamin k antagonists

## Abstract

The existing evidence on the use of non-vitamin K oral anticoagulants (NOACs) after transcatheter aortic valve replacement (TAVR) surgery is inconclusive and contradictory. Likewise, major society guidelines remain ambivalent about NOAC use around the time of TAVR. The objective of our meta-analysis was to assess the efficacy of NOACs in comparison to vitamin K antagonists (VKAs) in lowering complications following TAVR. An electronic literature search was conducted using MEDLINE, EMBASE, the international clinical trials database, and Cochrane Library. The primary endpoint was all-cause mortality, and the secondary endpoints included cardiovascular-related death, myocardial infarction, all-cause stroke, and various bleeding events. Forest plots were constructed for the pooled analysis of data. Subgroup and sensitivity analyses were also performed. No significant difference was noted between the NOAC and VKA groups with respect to the risk of all-cause mortality (14.14% vs. 20.28%; risk ratio (RR): 0.82; 95% confidence interval (CI): 0.61 to 1.10; p = 0.18), cardiovascular-related deaths (6.10% vs. 8.55%; RR: 1.02; 95% CI: 0.78 to 1.33; p = 0.91), myocardial infarction (1.52% vs. 2.51%; RR: 1.13; 95% CI: 0.50 to 2.56; p = 0.77), all-cause stroke (2.74% vs. 2.68%; RR: 1.02; 95% CI: 0.75 to 1.39; p = 0.89), major bleeding (7.74% vs. 9.62%; RR: 0.97; 95% CI: 0.58 to 1.61; p = 0.90), minor clinically relevant bleeding (14.33% vs. 13.73%; RR: 0.91; 95% CI: 0.67 to 1.24; p = 0.55), and other bleeding events. The risk of intracranial hemorrhage was lower in the NOAC group than in the VKA group (0.45% vs. 0.67%; RR: 0.61; 95% CI: 0.42 to 0.88; p = 0.008). Our study found NOACs to be non-inferior to VKAs in several outcomes, such as all-cause mortality, cardiovascular-related deaths, myocardial infarction, all-cause stroke, and bleeding events. In fact, NOACs were favored over VKAs, as the VKA group had a higher risk of developing intracranial hemorrhage.

## Introduction and background

Aortic stenosis (AS) is a condition that becomes increasingly severe over time and is marked by constriction of the aortic valve, especially in elderly individuals. Surgical aortic valve replacement (SAVR) has long been considered the most effective treatment for AS. In recent years, transcatheter aortic valve replacement (TAVR) has emerged as a groundbreaking and minimally invasive alternative for patients who are not well suited for SAVR because of factors such as advancing age, high surgical risk, and the presence of comorbidities [1,2]. TAVR involves the percutaneous insertion of a bioprosthetic valve through a catheter, typically delivered via the femoral artery or alternative access routes, directly into the diseased aortic valve [3,4]. However, TAVR is associated with adverse events such as life-threatening bleeding (LtB), stroke, and arrhythmia, especially atrial fibrillation (AF) [5]. Therefore, to prevent adverse cardiovascular outcomes, dual antiplatelet therapy (DAPT) and anticoagulants are generally used postoperatively in patients undergoing TAVR.

The utilization of direct oral anticoagulants (OACs) (DOACs) and non-vitamin K antagonist OACs (NOACs) in patients undergoing TAVR has gained significant attention in recent years. Multiple clinical trials and observational studies have shown that DOACs, including dabigatran, rivaroxaban, apixaban, and edoxaban, are equally effective as vitamin K antagonists (VKAs), such as warfarin, in preventing stroke and systemic embolism in patients with AF [2,6-8]. However, the existing data on the use of NOACs after TAVR surgery yields conflicting results, posing a challenge for healthcare providers seeking to establish a uniform approach in their medical practice. Therefore, our study aimed to assess the effectiveness of NOACs compared with VKAs in reducing complications after TAVR in patients.

## Review

Materials and methods

Literature review and meta-analysis were conducted using the Preferred Reporting Items for Systematic Reviews and Meta-Analyses (PRISMA) guidelines [9]. The study protocol was registered with the International Prospective Register of Systematic Reviews (PROSPERO) under the ID CRD42023443372.

Search Strategy

A comprehensive electronic literature search was performed using MEDLINE (accessible through PubMed), EMBASE, the international clinical trials database (www.clinicaltrials.gov), and Cochrane Library to screen for randomized controlled trials (RCTs) and observational studies examining the efficacy of NOACs compared to VKAs in patients who had undergone TAVR procedure (searches were made without limitations on language or time). In addition, we performed manual searches of reference lists to discover any other papers that were pertinent to our research.

We utilized pre-established Medical Subject Headings (MeSH) phrases in combination with the Boolean operators “AND” and “OR.” The search approach included the phrases “TAVI”, “TAVR”, “transcatheter aortic valve implantation”, “transcatheter aortic valve replacement”, “DOAC”, “NOAC”, “direct oral anticoagulants”, and “non-vitamin K antagonist oral anticoagulants”.

Study Selection and Eligibility Assessment

Population (P), intervention (I), control (C), and outcomes (O), following the PICOS format, were utilized for the inclusion criteria, with P being patients undergoing the TAVR procedure, I being patients receiving NOACs, C being the control group receiving VKAs, and O being several outcomes defined below. The inclusion criteria for the studies were the following: (1) both RCTs and observational studies, (2) studies involving patients who had undergone TAVR, (3) studies comparing NOACs to VKAs, and (4) studies reporting any safety outcome of interest. The following criteria were used to exclude studies: (1) studies categorized as letters to editors/commentaries, case reports, guidelines, literature reviews, systematic reviews, and meta-analyses; (2) studies involving patients with a history of coexisting conditions that have a high risk of bleeding; (3) studies involving patients with mechanical heart valve prostheses; (4) studies involving patients with any absolute indication for DAPT; (5) studies conducted on animal models; and (6) studies lacking sufficient clinical information relevant to the outcomes being investigated.

Two investigators (AG and LF) independently reviewed the titles and abstracts for eligibility and excluded duplicates and studies that did not meet the inclusion criteria. After the initial screening, we thoroughly examined the full text of the remaining articles to extract data, including information such as author, year, study design, follow-up period, number of patients, patient characteristics, and outcomes. Any disagreements were resolved through discussion between two independent reviewers to reach a consensus. No limitations were imposed on the sample size or follow-up duration.

Study Outcomes

The primary endpoint was all-cause mortality, which was defined according to the Vascular Academic Research Consortium-2 criteria [10]. The secondary endpoints included cardiovascular-related deaths, myocardial infarction, all-cause stroke, ischemic stroke, hemorrhagic stroke, LtB, major bleeding episodes, minor clinically relevant bleeding (MCRB) events, fatal bleeding, bleeding, intracranial hemorrhage, and systemic embolism. Bleeding criteria were defined according to those established by the Vascular Academic Research Consortium-2 and the Bleeding Academic Research Consortium [10,11].

Data Extraction and Quality Assessment

Data extraction was carried out autonomously by three researchers (MDT, MC, and FQA). If there was a disagreement, an impartial researcher (LF) assessed the data. The collected data from eligible studies encompassed the name of the primary author, the year of publication, the country of origin, the study's design, the size of the sample, and the specifics of the intervention. The quality of RCTs was assessed using the modified Cochrane Collaboration's risk of bias tool [12]. In contrast, the Newcastle-Ottawa scale was used to assess the quality of observational studies [13].

Statistical Analysis

The collected data were consolidated for meta-analysis by two authors (MC and AG) using Review Manager (RevMan) Version 5.4 (The Cochrane Collaboration, London, England, UK). Forest plots were created to assess binary data with 95% confidence intervals (CIs). The risk ratio (RR) was used as the measure of effect to evaluate binary outcomes. Sensitivity analysis, using hazard ratios (HRs), was conducted on the adjusted estimates for the primary outcome of all-cause mortality in observational studies. The threshold for statistical significance was set at a p-value of less than 0.05. A random-effects model was applied to all outcomes. Statistical heterogeneity was assessed using Higgins' I^2^ statistics, with values categorized as mild (25% to 50%), moderate (50% to 75%), and severe (>75%) heterogeneity. Subgroup and leave-one-out sensitivity analyses were performed to explore potential sources of heterogeneity. Funnel plots were visually inspected for any indication of publication bias. Additionally, trim-and-fill meta-analyses were conducted for outcomes where large studies were missing to assess potential publication bias and its impact on the results. R software version 4.4.1 (R Foundation for Statistical Computing, Vienna, Austria) was used for leave-one-out sensitivity analyses and trim-and-fill meta-analyses.

Results

Study Selection

After conducting a thorough search using several databases, a total of 3,451 articles that could be related were found. Out of these articles, 2,702 were eliminated after the initial evaluation. The articles not included in the initial screening were omitted because they had duplicate data, and their titles and abstracts did not fit the criteria for inclusion.

**Figure 1:**
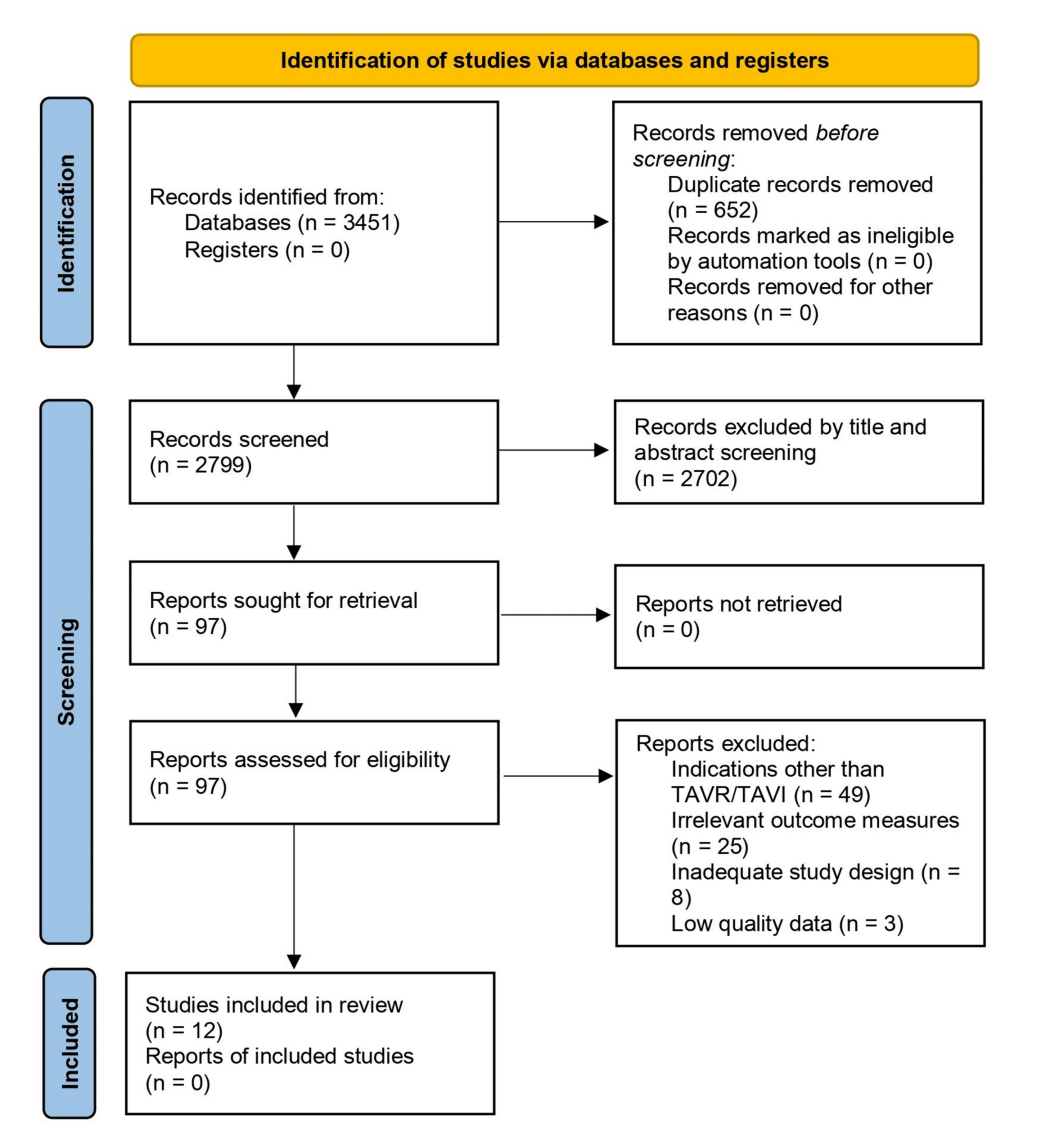
PRISMA flow chart for identification of studies included in the meta-analysis PRISMA: Preferred Reporting Items for Systematic Reviews and Meta-Analyses; TAVI: transcatheter aortic valve implantation; TAVR: transcatheter aortic valve replacement

The full texts of the remaining 97 articles were screened and rejected because of (1) indications other than TAVR/transcatheter aortic valve implantation (TAVI) (n = 49), (b) irrelevant outcome measures (n = 25), (c) inadequate study design (n = 8), and (d) low-quality data (n = 3). Finally, 12 studies were included in this meta-analysis [6,14-24]. The detailed steps of the literature search are presented in the PRISMA flowchart (Figure 1).

Study and Patient Characteristics

After exhaustive screening, we included 12 studies comprising 51,746 patients (12,762 patients in the intervention group receiving NOACs and 23,353 patients in the control group receiving VKA therapy). Table 1 shows the baseline characteristics of each study.

**Table 1:** Study characteristics and baseline values for patients in non-vitamin K antagonist oral anticoagulant (NOAC) vs vitamin K antagonist (VKA) groups RCT: randomized controlled trial; NR: not reported; CHA2DS2-VASc: congestive heart failure, hypertension, age ≥75 years (doubled), diabetes mellitus, prior stroke or transient ischemic attack or thromboembolism (doubled), vascular disease, age 65 to 74 years, sex category; SD: standard deviation

Author list, year of study	Study design	Sample size (total)	Sample size (NOAC/VKA)	Age in years (mean ± SD)		Number of male patients in %		CHA2DS2-VASc score (mean ± SD)		Hypertension (%)		Congestive heart failure (%)		Diabetes mellitus (%)		Coronary artery disease (%)	
				NOAC	VKA	NOAC	VKA	NOAC	VKA	NOAC	VKA	NOAC	VKA	NOAC	VKA	NOAC	VKA
Van Mieghem et al. 2021 [6]	RCT	1,426	713/713	82.1 ± 5.4	82.1 ± 5.5	51.3	53.6	4.5 ± 1.4	4.5 ± 1.3	90.7	92.1	82.9	86.8	37.9	36	41.1	41.7
Collet et al. 2022 [14]	RCT	451	223/228	82.3 ± 6.1	82.5 ± 6.3	45.9	47.9	4.7 ± 1.4	4.6 ± 1.4	82.1	79.4	52.5	49.6	27.8	31.6	NR	NR
Seeger et al. 2017 [15]	Observational	272	141/131	82.1 ± 53	80.5 ± 6.3	50.4	48.1	5.0 ± 1.2	4.9 ± 1.1	NR	NR	NR	NR	46	42	93	77
Jochheim et al. 2019 [16]	Observational	962	326/636	81.6 ± 6.7	81.1 ± 6.1	47.9	47.3	NR	NR	89.9	89.5	85.6	74.5	28.8	34.1	NR	NR
Butt et al. 2021 [17]	Observational	735	219/516	81.9 ± 5.2	81.3 ± 5.95	53.9	53.7	5.0 ± 1.4	4.9 ± 1.3	87.2	88.6	47.5	47.3	17.8	24.2	54.3	54.5
Didier et al. 2021 [18]	Observational	24,581	2,180/6,780	83.43 ± 5.93	82.95 ± 6.61	50.96	52.36	NR	NR	NR	NR	64.7	71	24.1	27.5	NR	NR
Geis et al. 2018 [19]	Observational	326	154/172	83.1 ± 5.3	83.0 ± 4.9	49	45	4.6 ± 1.2	4.8 ± 1.3	95	92	29	33	31	33	52	51
Kawashima et al. 2020 [20]	Observational	403	227/176	84.4 ± 4.7	84.3 ± 4.9	30.4	36.9	5.1 ± 1.0	5.2 ± 1.1	75.8	76.7	NR	NR	24.2	24.2	26	35.2
Kosmidou et al. 2019 [21]	Observational	933	155/778	NR	NR	NR	NR	NR	NR	NR	NR	NR	NR	NR	NR	NR	NR
Kalogeras et al. 2019 [22]	Observational	227	115/102	81.9 ± 6.3	82.5 ± 5.8	59.1	41.5	NR	NR	NR	NR	NR	NR	24.3	26.8	NR	NR
Tanawuttiwat et al. 2022 [23]	Observational	21,131	8,127/13,004	82.65 ± 6.67	82.95 ± 6.67	56.9	56.6	3.00 ± 1.48	3.0 ± 1.49	92.2	91.4	NR	NR	35.6	37.3	NR	NR
Mangner et al. 2019 [24]	Observational	598	182/117	80.35 ± 5.23	80 ± 4.50	48.9	47.9	5.65 ± 0.75	5.65 ± 0.75	96.2	99.1	NR	NR	42.3	42.2	39.2	47.0

Outcomes

Thirteen outcomes were reported in the current analysis.

**Figure 2:**
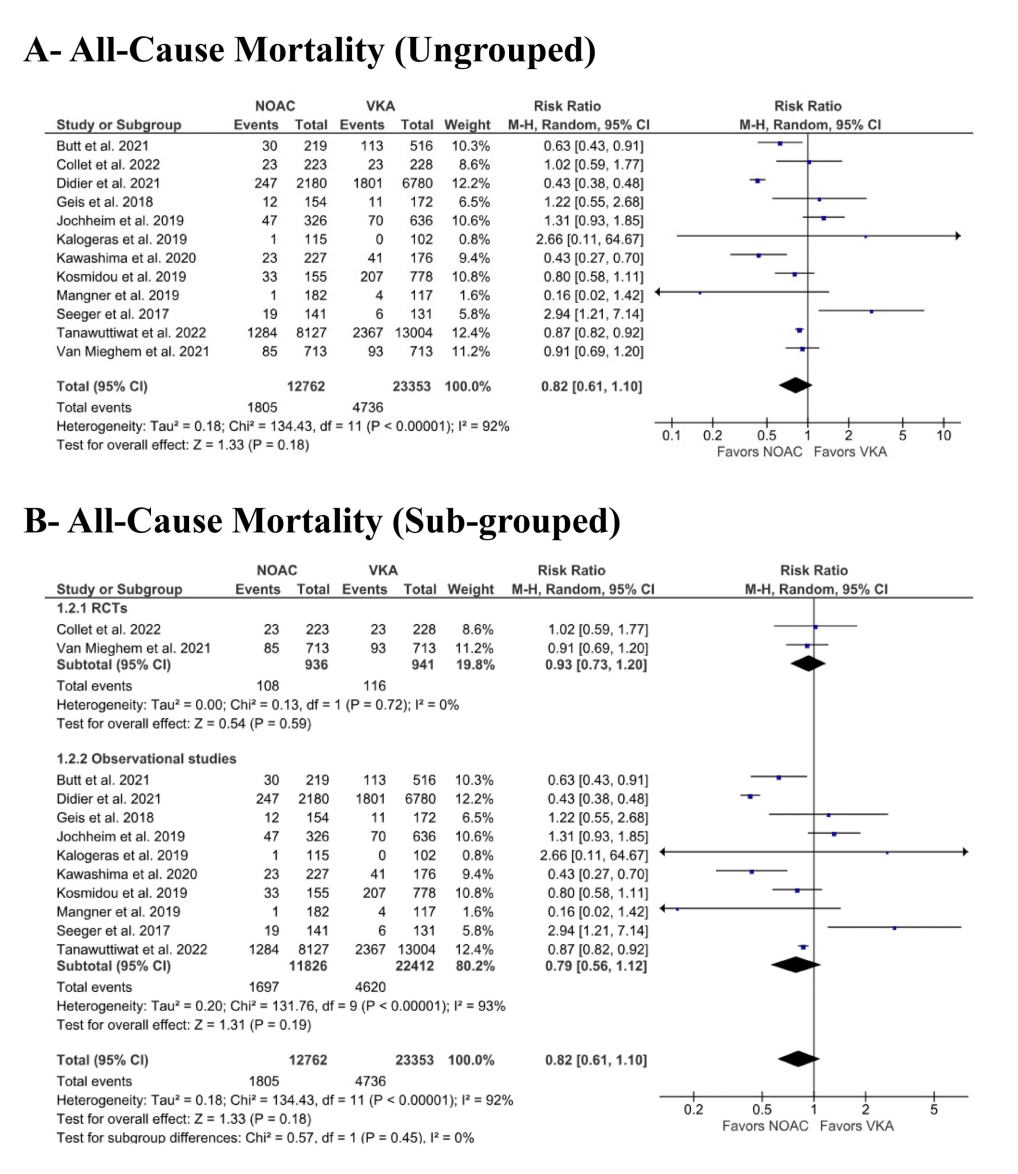
(A) Comparison of all-cause mortality between the NOAC and VKA groups; (B) comparison of all-cause mortality between the NOAC and VKA groups, subgrouped by RCTs and observational studies NOAC: non-vitamin K oral anticoagulant; VKA: vitamin K anticoagulant; RCT: randomized controlled trial; CI: confidence interval References [6,14-24]

All-cause mortality:* *All-cause mortality was reported in all included studies [6,14-24]. No significant difference was noted between the NOAC and VKA groups with respect to all-cause mortality (14.14% vs. 20.28%; RR: 0.82; 95% CI: 0.61 to 1.10; p = 0.18; I^2^ = 92%) (Figure 2A). As this study reported high heterogeneity, we divided our included studies based on their design (i.e., RCTs and observational studies) (Figure 2B). We achieved low heterogeneity in the RCT group (RR: 0.93; 95% CI: 0.73 to 1.20; p = 0.59; I^2^ = 0%). However, the observational study group continued to show high heterogeneity (RR: 0.79; 95% CI: 0.56 to 1.12; p = 0.19; I^2^ = 93%). In addition, heterogeneity did not decrease significantly with the leave-one-out sensitivity analysis. However, excluding Didier et al. [18] reduced heterogeneity to 65% without significantly affecting the effect size (RR: 0.88; 95% CI: 0.72 to 1.08) (Supplementary Material 1). Upon performing a sensitivity analysis of adjusted estimates for all-cause mortality from observational studies, the results showed no statistical difference between the NOAC and VKA groups (HR: 1.00: 95% CI: 0.76 to 1.31; p = 0.99) (Supplementary Material 2).

**Figure 3:**
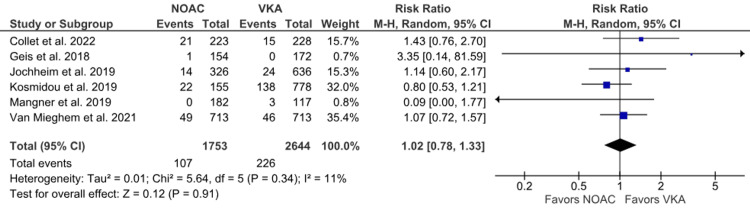
Comparison of cardiovascular-related deaths between the NOAC and VKA groups NOAC: non-vitamin K oral anticoagulant; VKA: vitamin K anticoagulant; CI: confidence interval References [6,14,16,19,21,24]

Cardiovascular-related deaths: Six studies reported cardiovascular-related deaths [6,14,16,19,21,24]. We observed that NOACs were not inferior to VKAs in terms of cardiovascular-related deaths (6.10% vs. 8.55%; RR: 1.02; 95% CI: 0.78 to 1.33; p = 0.91; I^2^ = 11%) (Figure 3). Low heterogeneity was observed between the studies.

**Figure 4:**
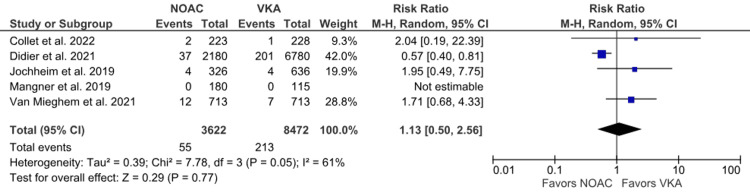
Comparison of myocardial infarction between the NOAC and VKA groups NOAC: non-vitamin K oral anticoagulant; VKA: vitamin K anticoagulant; CI: confidence interval References [6,14,16,18,24]

Myocardial infarction: The incidence of myocardial infarction was reported in five studies [6,14,16,18,24]. Our analysis showed that NOACs and VKAs did not significantly differ in the incidence of myocardial infarction (1.52% vs. 2.51%; RR: 1.13; 95% CI: 0.50 to 2.56; p = 0.77; I^2^ = 61%) (Figure 4). Moderate heterogeneity was also observed, for which we performed a leave-one-out sensitivity analysis. After excluding the study by Didier et al. [18], we observed that heterogeneity decreased to 0% with a slight change in effect size (RR: 1.81; 95% CI: 0.87 to 3.76) (Supplementary Material 3A).

**Figure 5:**
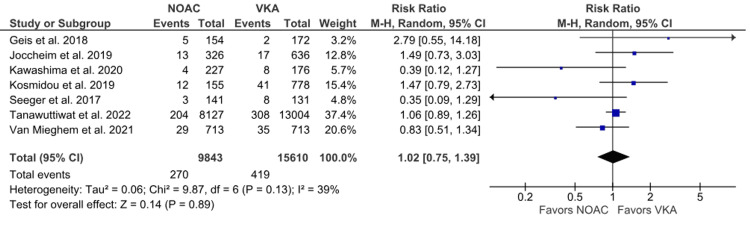
Comparison of all-cause stroke between the NOAC and VKA groups NOAC: non-vitamin K oral anticoagulant; VKA: vitamin K anticoagulant; CI: confidence interval References [6,15,16,19-21,23]

All-cause stroke: All-cause stroke outcomes were reported in seven studies [6,15,16,19-21,23]. The incidence of all-cause stroke was comparable between the groups. We did not observe any statistical difference between NOACs and VKAs (2.74% vs. 2.68%; RR: 1.02; 95% CI: 0.75 to 1.39; p = 0.89; I^2^ = 39%) (Figure 5). Mild heterogeneity was observed among studies, which did not reduce significantly by leave-one-out sensitivity analysis (Supplementary Material 3B).

**Figure 6:**
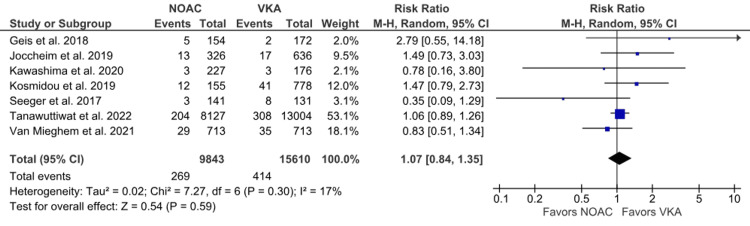
Comparison of ischemic stroke between the NOAC and VKA groups NOAC: non-vitamin K oral anticoagulant; VKA: vitamin K anticoagulant; CI: confidence interval References [6,15,16,19-21,23]

Ischemic stroke: The outcome of ischemic stroke was reported in seven studies [6,15,16,19-21,23]. We found no statistical difference in the incidence of ischemic stroke between the NOAC and VKA groups in comparison (2.73% vs. 2.65%; RR: 1.07; 95% CI: 0.84 to 1.35; p = 0.59; I^2^ = 17%) (Figure 6). Low heterogeneity was observed among studies.

**Figure 7:**
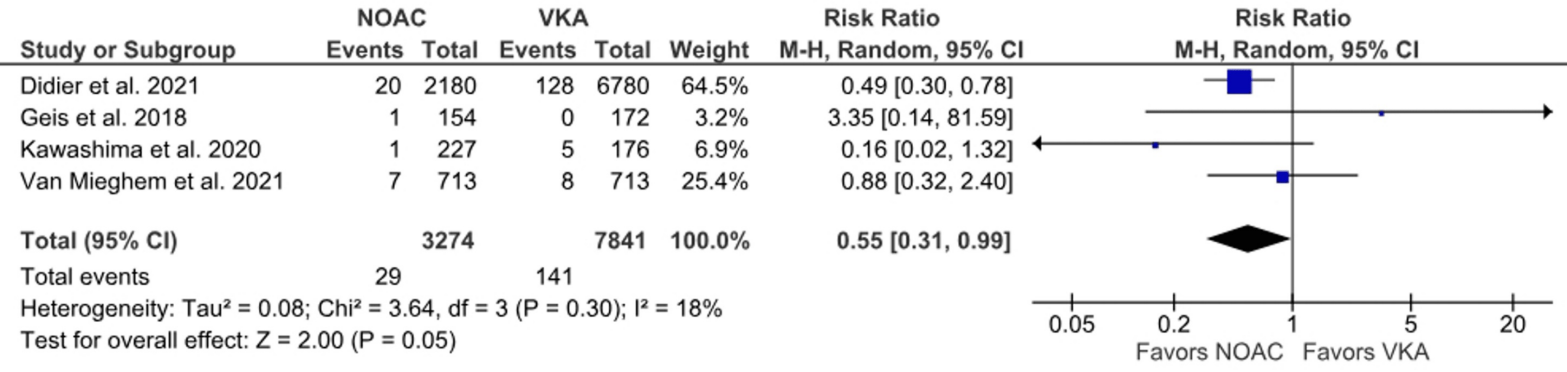
Comparison of hemorrhagic stroke between the NOAC and VKA groups NOAC: non-vitamin K oral anticoagulant; VKA: vitamin K anticoagulant; CI: confidence interval References [6,18-20]

Hemorrhagic stroke: Hemorrhagic stroke outcomes were reported in four studies [6,18-20]. The incidence of hemorrhagic stroke was higher in the VKA group, but no statistically significant difference was observed between the NOAC and VKA groups (0.89% vs. 1.80%; RR: 0.55; 95% CI: 0.31 to 0.99; p = 0.05; I^2^ = 18%) (Figure 7). Low heterogeneity was observed among the studies.

**Figure 8:**
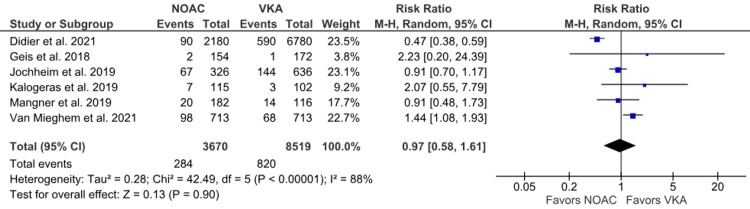
Comparison of major bleeding between the NOAC and VKA groups NOAC: non-vitamin K oral anticoagulant; VKA: vitamin K anticoagulant; CI: confidence interval References [6,16,18,19,22,24]

Major bleeding: Six studies reported major bleeding [6,16,18,19,22,24]. A higher incidence of major bleeding was noted in the VKA group than in the NOAC group; however, no statistically significant difference was observed between the two groups (7.74% vs. 9.62%; RR: 0.97; 95% CI: 0.58 to 1.61; p = 0.90; I^2^ = 88%) (Figure 8). A high heterogeneity was noted among the studies. On leave-one-out sensitivity analysis by excluding Didier et al. [18], heterogeneity was reduced to 43% with a slight change in effect size (RR: 1.14; 95% CI: 0.84 to 1.55) (Supplementary Material 3C).

**Figure 9:**
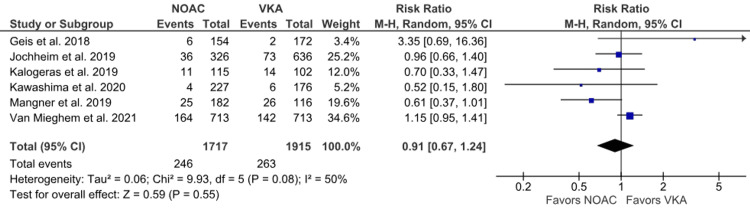
Comparison of minor clinically relevant bleeding (MCRB) between the NOAC and VKA groups NOAC: non-vitamin K oral anticoagulant; VKA: vitamin K anticoagulant; CI: confidence interval References [6,16,19,20,22,24]

MCRB: The MCRB outcome was reported in six studies [6,16,19,20,22,24]. A higher incidence of MCRB was observed in the NOAC group, but no statistical significance was observed between the NOAC and VKA groups (14.33% vs. 13.73%; RR: 0.91; 95% CI: 0.67 to 1.24; p = 0.55; I^2^ = 50%) (Figure 9). Moderate heterogeneity was observed among studies, which was reduced to 26% upon leaving out Mangner et al. [24] with no significant difference in effect size (RR: 1.03; 95% CI: 0.79 to 1.34) (Supplementary Material 4A).

**Figure 10:**
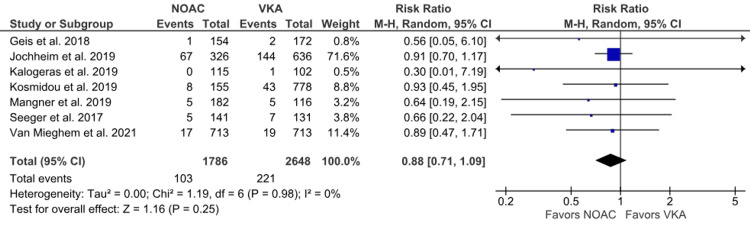
Comparison of life-threatening bleeding (LtB) between the NOAC and VKA groups NOAC: non-vitamin K oral anticoagulant; VKA: vitamin K anticoagulant; CI: confidence interval References [6,15,16,19,21,22,24]

LtB: Seven outcomes of LtB have been reported [6,15,16,19,21,22,24]. A higher incidence of LtB was noted in the VKA group than in the NOAC group. However, the results were not statistically significant between the NOAC and VKA groups (5.77% vs. 8.34%; RR: 0.88; 95% CI: 0.71 to 1.09; p = 0.25; I^2^ = 0%) (Figure 10). No heterogeneity was observed between the studies.

**Figure 11:**
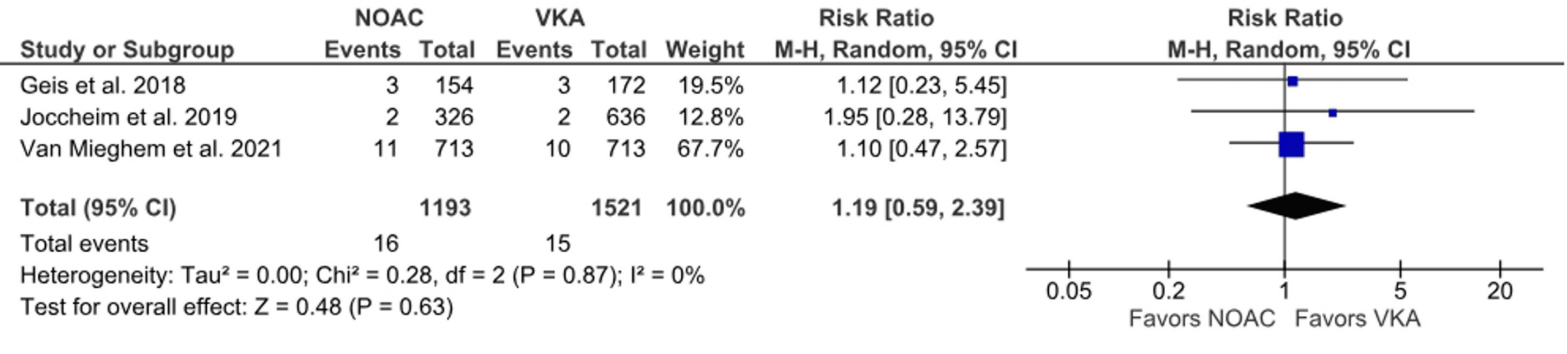
Comparison of fatal bleeding between the NOAC and VKA groups NOAC: non-vitamin K oral anticoagulant; VKA: vitamin K anticoagulant; CI: confidence interval References [6,16,19]

Fatal bleeding: Only three studies reported fatal bleeding as an outcome in their studies [6,16,19]. The incidence of fatal bleeding was higher in the NOAC group. However, no statistically significant difference was observed in our analysis between the NOAC and VKA groups (1.34% vs. 0.99%; RR: 1.19; 95% CI: 0.59 to 2.39; p = 0.63; I^2^ = 0%) (Figure 11). No heterogeneity was observed between the studies.

**Figure 12:**
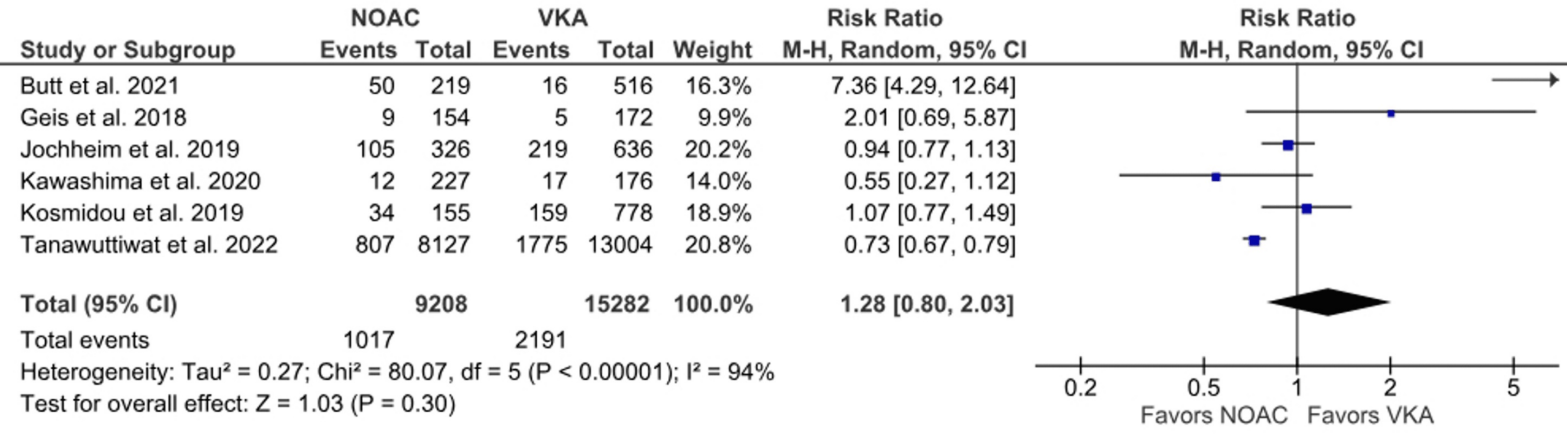
Comparison of any bleeding between the NOAC and VKA groups NOAC: non-vitamin K oral anticoagulant; VKA: vitamin K anticoagulant; CI: confidence interval References [16,17,19-21,23]

Any bleeding: Six studies reported any bleeding outcomes [16,17,19-21,23]. The incidence of AB was notably higher in the VKA group than in the NOAC group. No statistical significance was observed in the analysis between the two groups (11.04% vs. 14.34%; RR: 1.28; 95% CI: 0.80 to 2.03; p = 0.30; I^2^ = 94%) (Figure 12). High heterogeneity was noted among the studies, and the leave-one-out sensitivity analysis by excluding Butt et al. [17] slightly reduced heterogeneity to 71% with a minimal change in effect size (RR: 0.87; 95% CI: 0.69 to 1.10) (Supplementary Material 4B).

**Figure 13:**
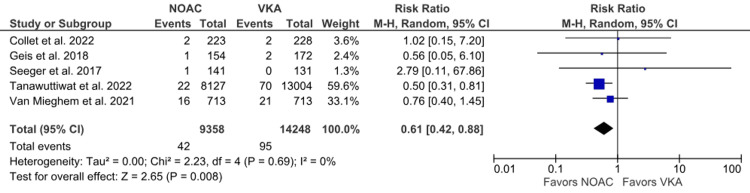
Comparison of intracranial hemorrhage between the NOAC and VKA groups NOAC: non-vitamin K oral anticoagulant; VKA: vitamin K anticoagulant; CI: confidence interval References [6,14,15,19,23]

Intracranial hemorrhage: Five studies reported intracranial hemorrhage as an outcome [6,14,15,19,23]. The incidence of intracranial hemorrhage was higher in the VKA group, and statistical significance was noted for lower relative risk in the NOAC group than in the VKA group (0.45% vs. 0.67%; RR: 0.61; 95% CI: 0.42 to 0.88; p = 0.008, I^2^ = 0%) (Figure 13). No heterogeneity was observed between the studies.

**Figure 14:**
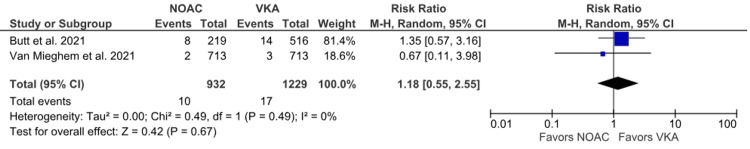
Comparison of systemic embolism between the NOAC and VKA groups NOAC: non-vitamin K oral anticoagulant; VKA: vitamin K anticoagulant; CI: confidence interval References [6,17]

Systemic embolism: Only two studies have reported systemic embolism as an outcome [6,17]. The incidence of systemic embolism was lesser in the NOAC group. No statistically significant difference was observed between the NOAC and VKA groups (1.07% vs. 1.38%; RR: 1.18; 95% CI: 0.55 to 2.55; p = 0.67; I^2^ = 0%) (Figure 14). No heterogeneity was observed between the studies.

Quality Assessment and Publication Bias

Quality assessment was performed using Cochrane's risk of bias tool (Supplementary Material 5) and the Newcastle-Ottawa scale (Supplementary Material 6) for RCTs and observational studies, respectively. Funnel plots were used to visually interpret publication bias (Supplementary Materials 7-9 ). All the plots were symmetrical, indicating no publication bias. Because of the symmetrical representation of funnel plots, we did not perform Egger's test or Begg's test. The trim-and-fill meta-analyses for cardiovascular-related deaths and fatal bleeding showed no differences in the results. However, non-significant differences were observed in the results for MCRB (RR: 0.95; 95% CI: 0.70 to 1.28) and LtB (RR: 0.89; 95% CI: 0.71 to 1.10) when one and two studies were added, respectively. Trim-and-fill analysis could not be performed for systemic embolism due to the inclusion of fewer than three studies.

Discussion

Our analysis demonstrated comparable risks for all-cause mortality, cardiovascular death, myocardial infarction, major/minor bleeding, LtB, fatal bleeding, ischemic stroke, or systemic embolism in post-TAVR patients taking NOACs and VKAs, irrespective of any pre-existing indications for anticoagulation. We also found a significantly increased risk of intracranial hemorrhage in the VKA group, favoring the use of NOACs over VKAs.

In 2019, the United States reported 72,991 TAVR procedures performed compared to 57,626 SAVR procedures [25]. The inclination toward biological valve prostheses increased by 40.1% [26]. Like any other transcatheter procedure, TAVR carries an unavoidable risk of thrombotic and bleeding events in both short-term and long-term time frames [27]. Anticoagulation agents play an important role in preventing these complications, both in the long and short terms. However, the American Heart Association (AHA)/American College of Cardiology (ACC) 2020 guidelines suggest no specific recommendation on the choice of antithrombotic therapy. The European Society of Cardiology (ESC)/European Association for Cardio-Thoracic Surgery (EACTS) 2021 guidelines suggest that routine use of OACs is not recommended for patients with no baseline indications for oral anticoagulation. Lifelong OAC is recommended for post-TAVR patients with underlying indications for chronic oral anticoagulation [27]. A direct comparison of the use of NOACs or VKAs in patients or those without other indications was not provided by either of the guidelines.

Contrary to previous meta-analyses that directly compared NOACs with VKAs, our study included the largest patient cohort ever assembled to compare similar treatment and control groups. This distinction arises from the inclusion of recently published observational studies that had not been included before. Previous meta-analyses by Liang et al., Ueyama et al., Ooi et al., and Lee et al. primarily focused on patient populations with specific indications for OAC use due to pre-existing AF [28-31]. Our study included patients irrespective of their existing OAC use to understand the broader context of post-TAVR physiology.

The POPular-TAVI trial conducted by Nijenhuis et al. focused on antithrombotic therapy post-TAVR. The study cohort that had an indication for OACs contained two groups: one receiving OACs (low-molecular-weight heparin (LMWH), VKAs, or NOACs) only and the other requiring OAC plus clopidogrel. OAC monotherapy has been found to be equal to or superior to OAC plus clopidogrel therapy [32]. This trial was a significant addition to the academic literature, but certain questions remain unanswered regarding the use of NOACs versus VKAs [33].

Several trials have been conducted to determine the optimal antithrombotic regimen of NOACs versus VKAs post-TAVR. The ATLANTIS study focused on systemic anticoagulation using a direct Xa inhibitor post-TAVR, irrespective of any indication for anticoagulation. In patients with non-valvular AF, apixaban was superior to both aspirin and VKAs [14]. ENVISAGE AF deals with prevalent or incident AF post-TAVR. Although edoxaban was found to be non-inferior in terms of all-cause mortality, myocardial infarction, and stroke, it showed a greater risk of major bleeding, especially in the gastrointestinal tract [34].

The ARISTOTLE trial provided valuable information regarding the safety and efficacy of apixaban in individuals with AF [35]. The findings of the ARISTOTLE trial aligned with those of the ENGAGE AF-TIMI 48 trial, which demonstrated similar levels of safety and effectiveness between edoxaban and warfarin. This suggests that NOACs could be a viable option for preventing thromboembolism in patients with AF and a history of valve surgery [36].

However, none of these studies specifically mentioned patients with a post-TAVR status. Guimarães et al. performed a post hoc analysis of the ARISTOTLE study to answer the question of NOACs vs. VKAs for bioprosthetic valves or prior to valve surgery; they were limited by the small sample size and less specific data on the post-TAVR status [37].

These studies underscore the diverse profiles of TAVR patients, from those with AF to those with symptomatic AS and intermediate operative risk, highlighting its necessity. The ongoing debate between NOACs and VKAs is a critical consideration, with ATLANTIS suggesting apixaban's superiority [14]. However, ENVISAGE AF raises concerns about edoxaban's bleeding risk [34].

Several observational studies over the years have discussed the use of NOACs versus VKAs, often preferring one over the other for different outcomes. For instance, Butt et al. conducted a study comparing the overall mortality rates of NOACs and VKAs, including subgroup analyses for dabigatran versus VKAs, rivaroxaban versus VKAs, and apixaban versus VKAs. Their results showed no significant difference in death rates between these groups [17]. Similarly, Didier et al., Kawashima et al., Tanawuttiwat et al., and Mangner et al. observed comparable mortality results in patients undergoing TAVR with concurrent AF [18,20,23,24]. Additionally, Jochheim et al. and Kalogeras et al., following one-year and two-year follow-ups, respectively, found no notable disparities in death rates between the NOAC and VKA groups [16]. Van Mieghem et al., Collet et al., Geis et al., and Kosmidou et al. also reported no significant difference in mortality between these anticoagulant therapies [6,14,19,21]. However, Seeger et al. reported increased mortality in the NOAC group [15]. To address these mixed findings, our subgroup analysis of nine observational studies revealed a non-significant difference between the two groups in the incidence of all-cause mortality.

As pointed out in the ENVISAGE trial, as one of the most important complications of NOAC use, major bleeding (including LtB) was also used to assess safety [34]. Although most studies that reported major bleeding showed no significant increase in the risk, Didier et al. also suggested a lower risk with the use of NOAC, and Van Mieghem et al. showed an increased risk of bleeding [6,18]. However, our analysis showed no difference in the risk of bleeding between the two groups.

In the present study, the risk of stroke (both hemorrhagic and ischemic) was the same in both groups. This finding is consistent with the outcomes of the ENVISAGE trial of NOACs [34]. Compared with the findings for non-valvular AF in the ARISTOTLE trial, we found similar results for ischemic stroke; however, the incidence of hemorrhagic stroke showed no difference in our analysis compared to the significant decrease found in the ARISTOTLE trial [35]. Another interesting finding in our analysis was an increase in the incidence of intracranial hemorrhage in the VKA group from the pooling of six studies that favored NOACs, which was not mentioned in any previous meta-analyses.

There has been an increase in discussions regarding cardiovascular-related deaths and myocardial infarction after TAVR or TAVR/TAVI. For this, anticoagulants reduce the risk of thrombus formation on or around the implanted valve, which could lead to acute myocardial infarction if the thrombi embolize and block coronary arteries. Didier et al. reported a decrease in myocardial infarction with the use of NOACs [18]. However, our pooled analysis of five studies revealed no differences in cardiovascular death or myocardial infarction between the groups.

Given the increasing preference for TAVR over SAVR in patients with AS, it is critical to identify the optimal anticoagulation strategy to enhance patient outcomes. Previous studies indicate that other therapies, such as DOACs, also have proven efficacy and a favorable safety profile [38]. Therefore, to comprehensively compare the safety and efficacy of these anticoagulant therapies, future research, particularly large-scale RCTs with multiple treatment arms and extensive network meta-analyses, is essential. These studies could reveal the optimal anticoagulation regimen, potentially leading to improved clinical guidelines and better patient outcomes following TAVR.

Limitations

Our meta-analysis has several limitations. First, most of the included studies were observational and retrospective in nature, which is a source of heterogeneity. Moreover, the RRs presented in our study were not adjusted for confounding variables, which may have affected our results. However, a sensitivity analysis for adjusted estimates of all-cause mortality gave similar results. Second, the treatment arm in our study included all four currently used NOACs at varying doses, combined in a single arm rather than exploring the effects of each drug individually. While a subgroup analysis for each specific drug, including both low and high doses, was intended, it could not be performed due to the lack of sufficient studies with such specific data. Our study did not differentiate between patients with or without specific indications for OAC use. Although most patients in our study had AF as an indication for OAC use, we also included studies with other indications, such as venous thromboembolism and hypercoagulability. Lastly, the concomitant use of antiplatelets varied across the studies, with most of the studies reporting the use of single antiplatelet therapy or DAPT during the early post-TAVR period, typically up to three months. This heterogeneity within the patient population should be considered when interpreting results.

## Conclusions

Our comprehensive meta-analysis highlights the use of NOACs as a viable therapeutic option for managing patients who have undergone TAVR. Our study found NOACs to be non-inferior to VKAs in several outcomes, such as all-cause mortality, cardiovascular-related deaths, myocardial infarction, all-cause stroke, and bleeding events. Our study favored the use of NOACs over VKAs, as VKAs have a higher risk of developing intracranial hemorrhage. Further large-scale RCTs are needed in these patients, preferably with multiple control arms, to compare various therapeutic options for physicians to aptly decide the best available management option for their own practice.

## Figures and Tables

**Figure 15:**
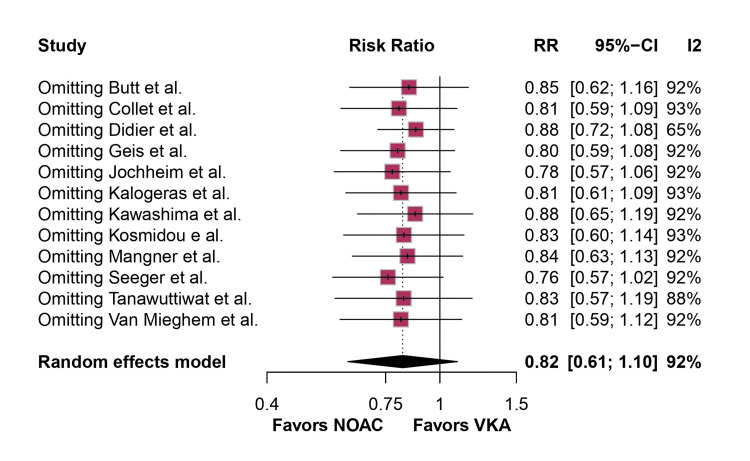
Leave-one-out sensitivity analysis for all-cause mortality NOAC: non-vitamin K oral anticoagulant; VKA: vitamin K anticoagulant References [6,14-24]

**Figure 16:**
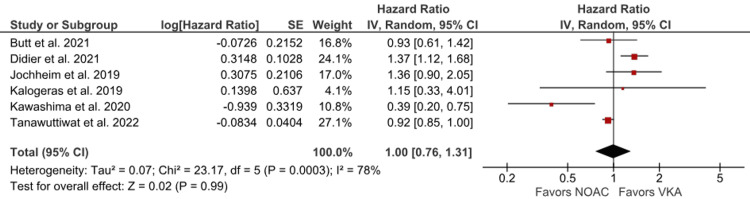
Sensitivity analysis for adjusted estimates for all-cause mortality among observational studies NOAC: non-vitamin K oral anticoagulant; VKA: vitamin K anticoagulant; CI: confidence interval; SE: standard error References [16-18,20,22,23]

**Figure 17:**
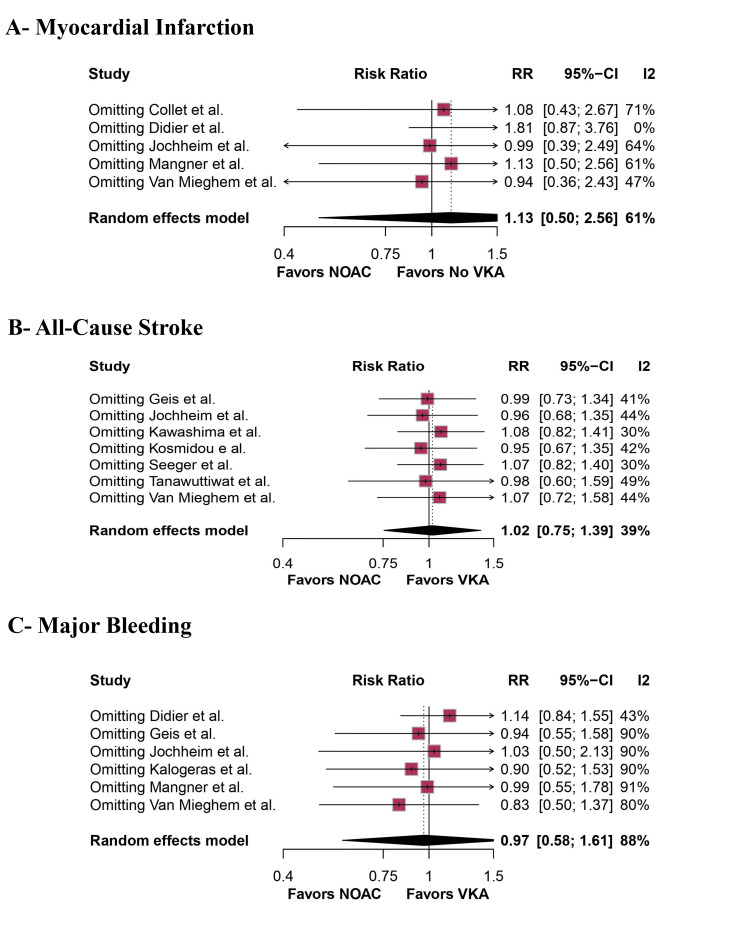
Leave-one-out sensitivity analysis for (A) myocardial infarction, (B) all-cause stroke, and (C) major bleeding NOAC: non-vitamin K oral anticoagulant; VKA: vitamin K anticoagulant References [6,14-16,18-24]

**Figure 18:**
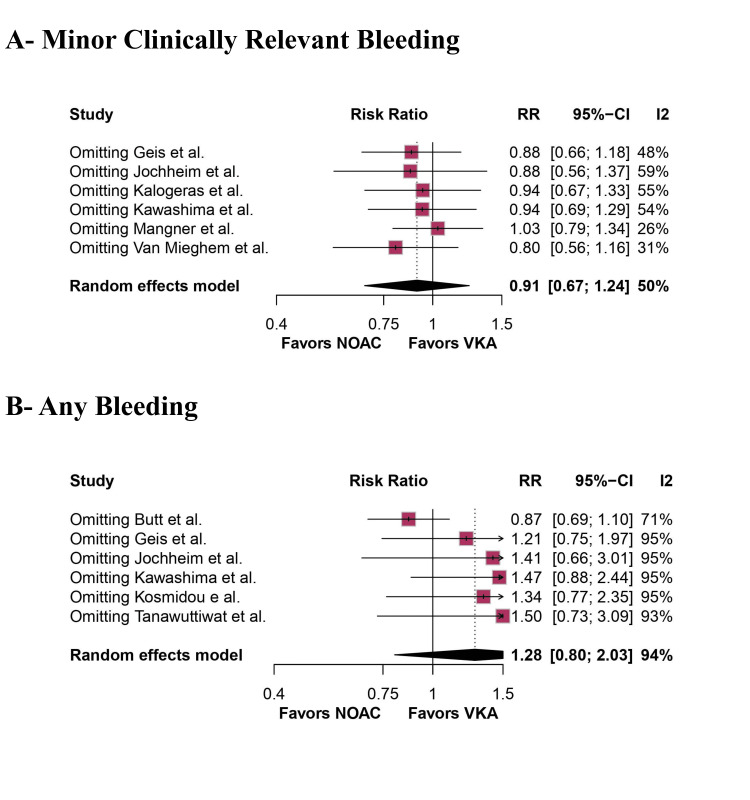
Leave-one-out sensitivity analysis for (A) minor clinically relevant bleeding and (B) any bleeding NOAC: non-vitamin K oral anticoagulant; VKA: vitamin K anticoagulant References [6,16,17,19-24]

**Figure 19:**
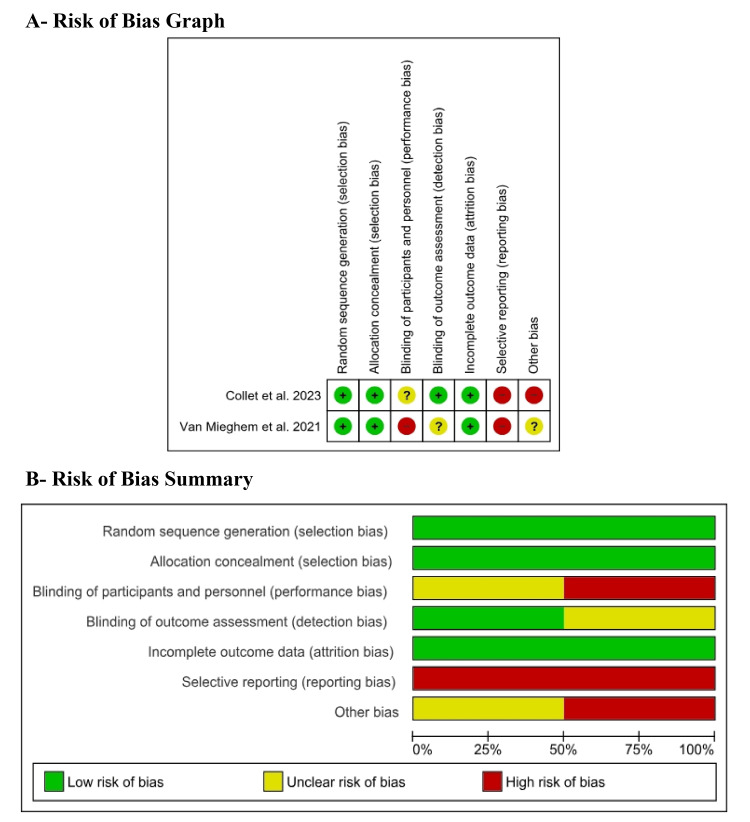
(A) Risk of bias graph; (B) risk of bias summary for randomized controlled trials using Cochrane’s risk of bias tool References [6,14]

**Figure 20:**
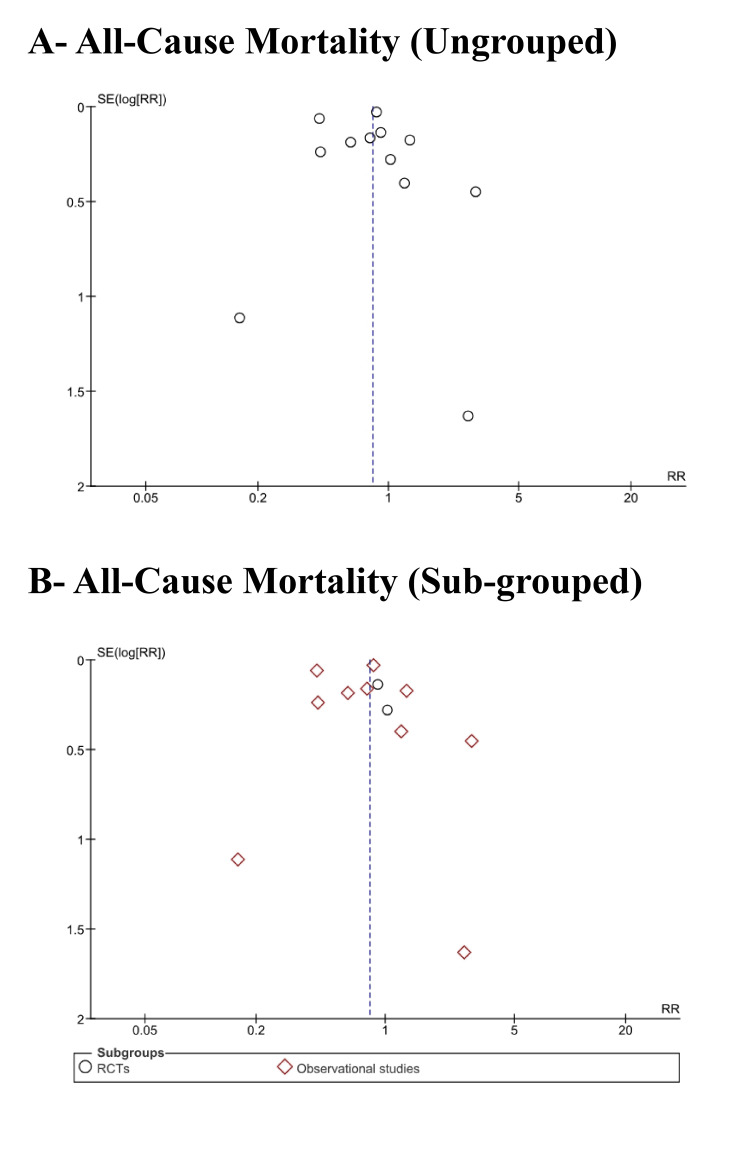
Funnel plot for the assessment of publication bias in (A) all-cause mortality and (B) all-cause mortality, subgrouped by RCTs and observational studies SE: standard error; RCT: randomized controlled trial

**Figure 21:**
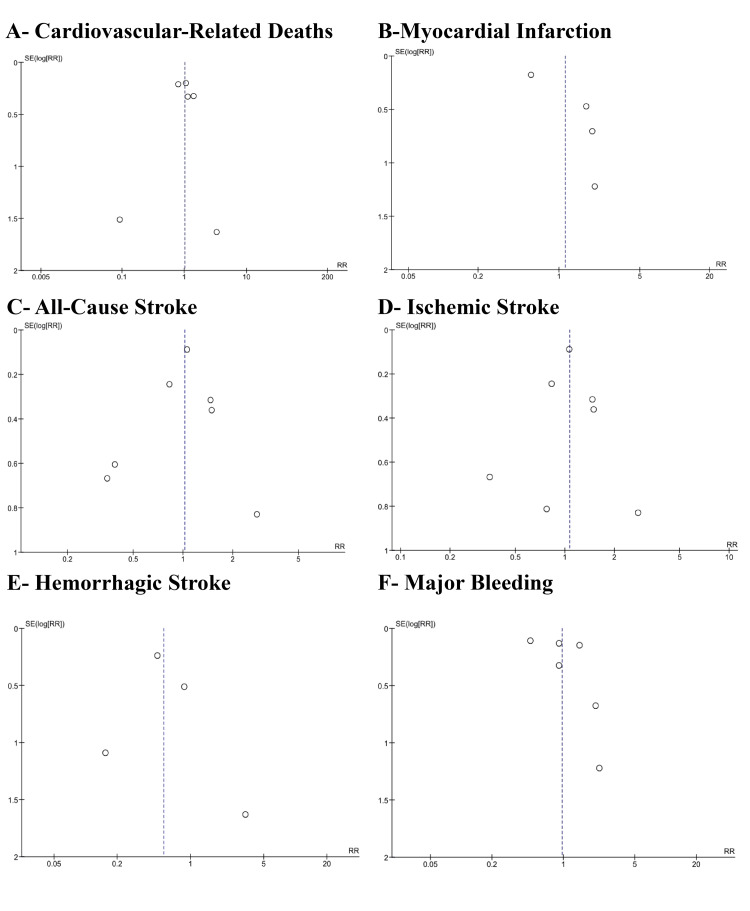
Funnel plots for the assessment of publication bias in (A) cardiovascular-related deaths, (B) myocardial infarction, (C) all-cause stroke, (D) ischemic stroke, (E) hemorrhagic stroke, and (F) major bleeding SE: standard error

**Figure 22:**
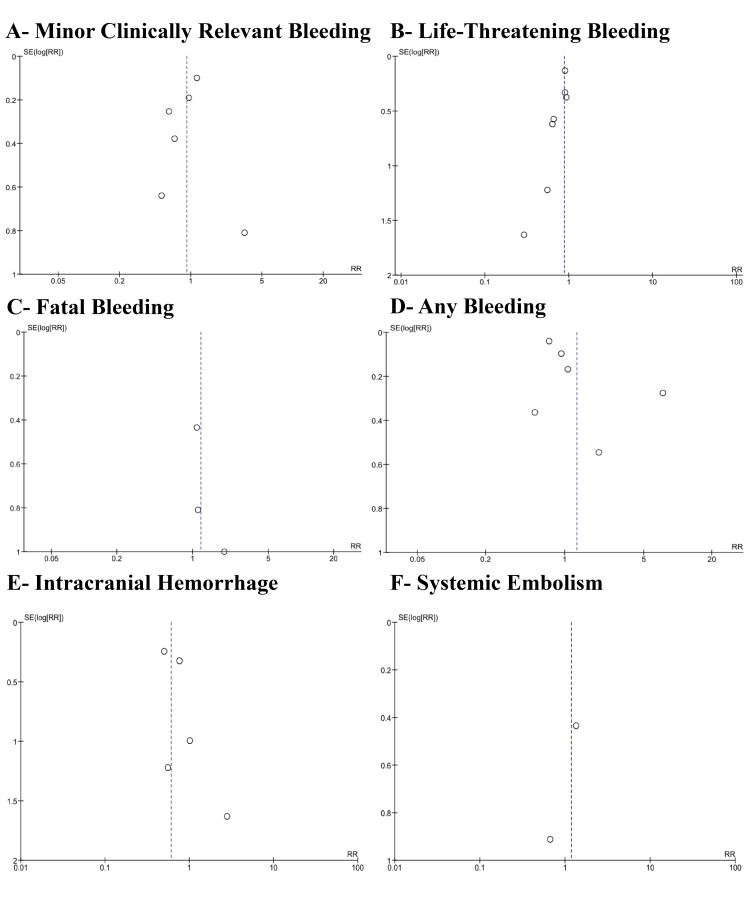
Funnel plots for the assessment of publication bias in (A) minor clinically relevant bleeding (MCRB), (B) life-threatening bleeding (LtB), (C) fatal bleeding, (D) any bleeding, (E) intracranial hemorrhage, and (F) systemic embolism SE: standard error

## Appendices

Supplementary Material 1

Supplementary Material 2

Supplementary Material 3

Supplementary Material 4

Supplementary Material 5

Supplementary Material 6

**Table 2:** Risk of bias assessment of observational studies using Newcastle-Ottawa Scale (NOS) The above sections in the table were rated per study, and the bias was assessed as low bias risk (8-9 points), moderate bias risk (5-7 points), and high bias risk (0-4 points)

Study	Selection (score)				Comparability	Outcome			Quality score
	Representativeness of the exposed cohort	Selection of the non-exposed cohort	Ascertainment of exposure	Demonstration that the current outcome of interest was not present at the start of the study	Comparability of cohorts based on the analysis or design	Assessment of outcome	Was follow-up long enough for outcomes to occur	Adequacy of follow-up of cohorts	
Seeger et al. 2017 [15]	1	1	0	1	2	1	0	1	7
Joccheim et al. 2019 [16]	1	1	1	1	2	1	0	1	8
Butt et al. 2021 [17]	1	1	1	1	1	1	1	1	8
Didier et al. 2017 [18]	1	1	1	1	2	1	1	1	9
Geis et al. 2018 [19]	1	1	0	1	2	1	1	1	8
Kawashima et al. 2020 [20]	1	1	1	1	2	1	1	1	9
Kosmidou et al. 2019 [21]	1	1	1	1	2	1	1	1	9
Kalogeras et al. 2019 [22]	1	1	0	1	2	1	0	1	7
Tanawuttiwat et al. 2022 [23]	1	1	1	1	2	1	0	1	8
Mangner et al. 2019 [24]	1	1	1	1	1	1	1	0	7

Supplementary Material 7 

Supplementary Material 8 

Supplementary Material 9 
